# New Functional Ingredients Based on Microencapsulation of Aqueous Anthocyanin-Rich Extracts Derived from Black Rice (*Oryza sativa* L.)

**DOI:** 10.3390/molecules24183389

**Published:** 2019-09-18

**Authors:** Iuliana Aprodu, Ștefania Adelina Milea, Roxana-Mădălina Anghel, Elena Enachi, Vasilica Barbu, Oana Crăciunescu, Gabriela Râpeanu, Gabriela Elena Bahrim, Anca Oancea, Nicoleta Stănciuc

**Affiliations:** 1 Faculty of Food Science and Engineering, Dunărea de Jos University of Galati, Romania, 111, Domnească Street, 800201 Galati, Romaniaadelina.milea@ugal.ro (Ș.A.M.); anghelroxanaro@yahoo.com (R.-M.A.); elena.ionita@ugal.ro (E.E.); vasilica.barbu@ugal.ro (V.B.);; 2 National Institute of Research and Development for Biological Sciences, 296, Splaiul Independentei, 060031 Bucharest, Romania; oana_craciunescu2009@yahoo.com (O.C.); oancea.anca@gmail.com (A.O.)

**Keywords:** anthocyanins, black rice, encapsulation, in silico, added value

## Abstract

The aqueous anthocyanin-rich extract derived from black rice (*Oryza sativa* L.) was encapsulated by freeze drying using milk proteins and peptides as coating materials. The molecular modelling approach indicated that all major casein fractions and whey proteins were able to bind at least one anthocyanin molecule. The hydrophobic interactions and hydrogen bonding across the interfaces appeared to be mainly responsible for the stabilizations of the complexes formed between the coating material and bioactive compounds. Two dark purple colored powders, differentiated by the ratio of the encapsulation materials used, rich in phytochemicals were obtained, with an encapsulation efficiency of up to 99%. The powders were tested for antioxidant activity, cytocompatibility, and thermal stability. The morphological structure of the powders highlighted the presence of encapsulated anthocyanins. Both powders showed a remarkable antioxidant activity of about 46 mM Trolox/g D.W., and cytocompatibility on the L929 fibroblast culture. At certain concentrations, both powders stimulated cell proliferation. The powders showed a good thermal stability between 75 and 100 °C for 15 min. The powders were tested in a food model system and checked for stability of phytochemicals during storage. The added value of the powders was demonstrated throughout the antioxidant activity, which remained unchanged during storage.

## 1. Introduction

Rice (*Oryza sativa* L.), as one of the most important staple crops, provides energy for almost half of the world’s population [1]. Most of the phytochemicals are present in the bran layer and embryo fraction [2]. Whole rice grains are hypothesized to contribute positively to human health due to their polyphenols, minerals, fiber, vitamins, and other phytochemicals [3,4], which may influence biological functions individually or synergistically [1]. The pigmentation of black rice samples is due to the accumulation of anthocyanins and proanthocyanidins in rice bran. Two major anthocyanins found in black rice are cyanidin-3-*O*-glucoside (C3G) and peonidin-3-*O*-glucoside (P3G) [5]. There are several reports suggesting that the consumption of pigmented rice can promote a decrease of oxidative stress, prevent inflammation, and reduce the risk of developing chronic diseases, like cardiovascular disease, type 2 diabetes, and some forms of cancer [6,7]. Pigmented rice has some other antioxidant compounds, such as flavones, proanthocyanidins, and phenolic acids, which contribute to its healthy nutritional profile [6,8]. It is well known that anthocyanins are a class of flavonoids, highly reactive and strongly subjected to degradation [8]. The anthocyanins’ stability highly depends on environmental and chemical factors, such as pH, metal ions, exposure to light and UV, temperature, oxygen, and enzymatic activity [9]. Consequently, due to their low stability during processing and storage, the direct use of these compounds in food formulation, especially in aqueous systems, is challenging [10].

Microencapsulation has proven to be an effective approach to improve the protection and stability of these compounds. Encapsulation is a technique used to entrap and protect an active agent within another substance, called the wall material or coating agent, working as a carrier and/or stabilizing agent [8]. Microencapsulation can be considered as a particularly fitting solution when it comes to antioxidant compounds, to protect the bioactive agents from adverse environmental conditions [11]. Therefore, the encapsulation of anthocyanins may provide alternatives for synthetic dyes because of their attractive bright color and water solubility, which allows them to be incorporated into foods [12], enhancing their possible health-promoting effects in humans. Milk proteins are of high value from a technological point of view, as well as for their beneficial physiological effects. It is well known that milk proteins, such as whey proteins and casein, have already been used in many studies for the encapsulation of bioactive compounds. 

Black rice anthocyanins are promising antioxidant agents that promote good health, and they can be used as functional food ingredients and nutraceuticals. However, few studies have focused on the encapsulation of black rice anthocyanins. Therefore, the objectives of the present work were to obtain anthocyanin-rich extracts from black rice, from the perspectives of microencapsulation. The extraction was performed in hot water (75 °C) for 12 h, followed by extract characterization and microencapsulation. For this purpose, the coating agents (whey protein isolate—WPI, whey protein hydrolysates—WPH, and casein—CN) were hydrated and dissolved into the black rice water extract, followed by freeze drying, to obtain stable anthocyanins powder. Prior microencapsulation, the in silico approach was used to check the interaction between milk proteins and major anthocyanins found in black rice, namely C3G and P3G, in order to characterize in great detail the protein–anthocyanin complexes, whereas the docking models were carefully analyzed in terms of interaction particularities. The obtained powders were characterized in terms of microencapsulation efficiency, phytochemical content, antioxidant activity, morphology, cytocompatibility, and thermal stability. The powders were then tested as functional ingredients in a pastry model food (cream for cakes), whereas the stability of phytochemicals and antioxidant activity during the accelerated storage test was also estimated.

## 2. Results

### 2.1. Characterization of the Polyphenolic Extract 

In the present work, an aqueous extract from black rice was obtained, with the aim of characterizing and testing different matrices for microencapsulation, in order to obtain a stable anthocyanin-rich ingredient for the production of functional foods or food supplements. The aqueous extract was analyzed for phytochemicals content and antioxidant activity. The total monomeric anthocyanins content (TAC) was 0.14 ± 0.01 mg C3G/g FW, a total flavonoids content (TFC) of 1.29 ± 0.04 CE mg/g FW, and a total polyphenolic content (TPC) of 1.71 ± 0.14 mg GAE/g FW. The extract showed a remarkable inhibition of 91.62 ± 0.24%, corresponding to 2,2-diphenyl-1-picrylhydrazyl (DPHH) scavenging capacity of 13.80 ± 0.82 mMol Trolox/g DW. Shao et al. [1] analyzed comparatively different varieties of rice and reported TFC values for red and black rice grains varying from 162.86 to 415.10 mg CE/100 g, whereas TAC was dependent on the genotypes, which differed from 0.058 to 2.54 mg/g in red rice, and from 0.015 to 1.41 mg/g in black rice, respectively. 

### 2.2. Microencapsulation Efficiency and Characterization of the Powders 

The anthocyanins extract encapsulating procedure involved the use, as encapsulation materials, of two encapsulation coatings ratio, differentiated by the casein concentration. Therefore, in the first experimental variant (further called variant 1), a ratio of WPI, WPH, and CN of 2:1:0.2 (*w:w:w*) was used, while in the second experimental version (further called variant 2) a ratio of 2:1:0.1 (*w:w:w*) was used. The experimental results indicated that the microencapsulation efficiency of anthocyanins in variant 1 was 98.58 ± 0.25% and 97.31 ± 0.66% in variant 2. Santos et al. [13] suggested that most physical encapsulation technologies can give a loading capacity as high as 99.0%. These authors suggested an encapsulation efficiency of 98.67% for anthocyanin extract from jabuticaba skins by using ionic gelification. Stănciuc et al. [14] used whey protein isolate and two different polysaccharides (acacia gum and pectin) to encapsulate grape anthocyanins by coacervation and freeze-drying, with an encapsulating efficiency between 94% and 99%. In the present study, the microencapsulated powders showed a TAC of 2.95 ± 0.12 mg C3G/g DW and 1.76 ± 0.09 mg C3G/g DW in variants 1 and 2, whereas TFC were 0.31 ± 0.001 and 0.40 ± 0.04 mg QE/g DW, respectively. Both variants showed a similar TPC of 1.96 ± 0.31 and 1.98 ± 0.2 mg GAE/g DW and antioxidant activity of 45.47 ± 4.10 and 46.13 ± 1.01 mM Trolox/g DW.

Rawel et al. [15] suggested that the anthocyanins’ hydroxyl group could interact with the amide carbonyl of the peptide backbone by hydrogen bonding. Both whey proteins and caseins have a high level of proline, which offer sites for anthocyanin binding [16]. Considering the experimental set-up used in the present study, it was assumed that the coating material interacted with C3G and P3G from black rice extract, facilitating microencapsulation. The experimental results were therefore complemented with an in-silico approach. Atomic level details on the interaction between the protein molecules found in the coating mixtures and anthocyanins were collected after performing docking tests.

Caseins are organized in milk into micelles, which are large raspberry-like colloidal particles with diameters ranging from 50 to 600 nm [17]. The surface of the micelles is coated with κ-casein, whereas the Ca-sensitive caseins are located toward the core of the sub-micelles. Caseins are aggregated through hydrogen bonds, and hydrophobic and electrostatic interactions [17]. The integrity of the casein micelle is reduced while processing to get the casein powders, leading to the release of important amounts of non-micellar casein [18]. Therefore, the molecular models of the principal casein fractions (αS1CN, αS2CN, βCN, and kCN) were used in addition to the main whey proteins (αLA and βLG) as receptors for anthocyanins binding. As indicated in Table 1, the interaction energy and contact area varied with the molecular assembly. Except for βCN, the contact surface was larger for complexes involving C3G as a ligand compared to P3G. No important variation of the binding strength between αLA and the two types of ligands was observed, although the contact area was significantly higher in the case of αLA–C3G (560.70 Å2), compared to the αLA–P3G complex (487.60 Å2). The αS1CN, βCN, and kCN appeared to bind tighter to the P3G molecule (interaction energy values of −219.83, −232.18, and −200.86 kJ/mol, respectively, lower compared to the corresponding complexes involving C3G), whereas αS2CN and βLG exhibited better affinity towards C3G (interaction energy values of −187.69 and −197.49 kJ/mol, respectively, lower compared to the corresponding complexes involving P3G). The hydrophobic interactions are mainly responsible for anthocyanins binding by all tested milk proteins. This observation is also supported by the negative values of the solvation free energy gain upon assembly formation (ΔG^int^), which correspond to hydrophobic interfaces, or positive protein affinity. In addition, particular attention was given to the hydrogen bonds (Hb), because the ΔG^int^ values do not include the effect of satisfied hydrogen bonds across the interfaces. In fact, various 2.10 to 3.30 Å long hydrogen bonds were found to stabilize the milk protein–anthocyanin complexes. The hydrogen bonds appear to play important roles at the protein–anthocyanins interfaces. 

**Figure 1:**
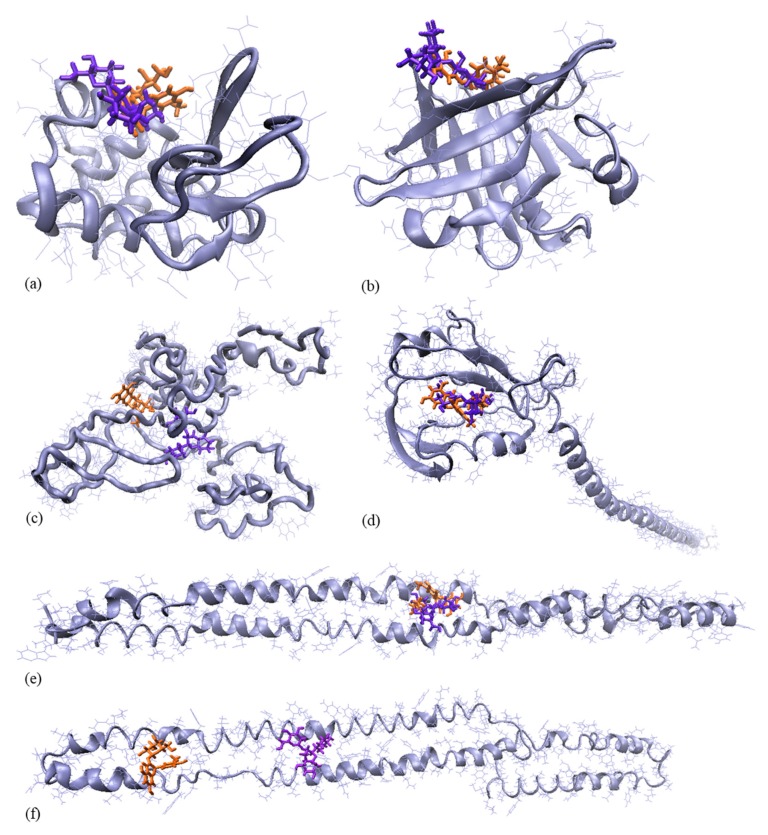
Single molecule level details on binding of cyanidin-3-*O*-glucoside (violet) and peonidin-3-*O*-glucoside (orange to the α-LA (**a**), β-LG (**b**), βCN (**c**), kCN (**d**), αS1CN (**e**), and αS2CN (**f**). Images were prepared using VMD software. The proteins are represented with ice blue in new cartoon style, while anthocyanins are represented in licorice style.

For instance, in the case of the kCN–C3G complex, the hydroxyl group at 6C of the glucose moiety from the anthocyanin structure is connected through three different Hb to Lys^107^, Thr^154^, and Glu^175^. The hydrogen bonding network across the βLG–P3G interface involves both the aglycon (the two hydroxyl groups of the aromatic ring bonded to the O-containing heterocyclic ring are connected through three Hb to Asp^85^ and Ala^86^) and the sugar moiety (the hydroxyl of 2C from the glucose structure is linked by Gln^108^ and Asn^109^). Finally, in the αLA–C3G complex, a double Hb connects one hydroxyl group substituted to the phenyl ring of the aglycon by Thr^33^, whereas the glucosyl unit is hydrogen bonded with Asn^102^ and Ala^106^. Positive free energy of assembly dissociation (ΔG^diss^) were estimated for all investigated milk protein–anthocyanin complexes, suggesting that they are thermodynamically stable. Regardless of the protein, the ΔG^diss^ values were higher for the C3G-involving assemblies (Table 1), indicating that the external forces required to dissociate the complexes need to be stronger compared to the corresponding complexes with P3G. The two anthocyanins share the same binding sites on the protein surface (Table 1), indicating that each receptor can bind one anthocyanin molecule. The exceptions concern the αS2CN and βCN, which are able to accommodate at the same time the two major anthocyanins from black rice (Figure 1). 

In the case of αS2CN, different sets of residues are involved in establishing hydrophobic contacts with C3G (Leu^114^, Leu^176^, Asn^177^, Leu^179^, Ile^182^, Ser^183^, Gln^187^) and P3G (Leu^121^, Pro^123^, Asp^125^, Val^127^, Arg^129^, Asn^130^, Val^154^, Glu^157^, Ser^158^). On the other hand, C3G and P3G are able to penetrate two different hydrophobic cavities of βCN, which share opposite sides of Glu^106^ and Val^107^ residues.

In agreement with the observation of Viljanen et al. [15], the present results indicated that the anthocyanins’ binding sites of βCN and kCN are rich in Pro residues. As indicated by the contact maps of the βCN-based assemblies, Pro^105^, Pro^165^, and Pro^167^ are involved in hydrophobic interactions with the C3G, whereas Pro^76^ and Pro^78^ are in contact with two hydroxyl groups substituted to the flavylium from the P3G molecule. In the case of the kCN-based complexes, Pro^120^ and Pro^171^ residues interfaced both anthocyanins considered in the docking study, in addition to the hydrophobic contacts established by Pro^177^ and Pro^105^ with the benzopyrylium cation from the C3G and P3G molecules, respectively.

### 2.3. Morphological Structure of the Microencapsulated Powders

**Figure 2:**
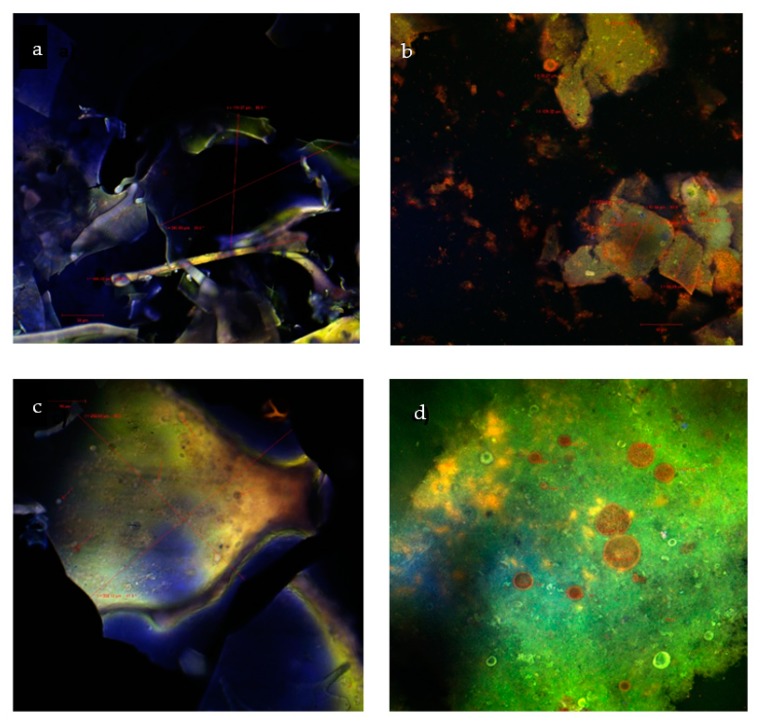
Confocal laser scanning microscopy images of the native microencapsulated powders: variant 1—native state (**a**) and with fluorophores (**b**); variant 2—in native state (**c**) and with fluorophores (**d**).

Figure 2a,c provide information about the shape of the native powders without any fluorophores. 

No significant differences between variant 1 and variant 2 were observed, the formations being visualized as fine scales with an autofluorescence in a broad spectral range. For variant 2, it was possible to detect, even in the native variant, some spherical particles formed after the encapsulation, inside of which smaller spherosomes were observed, hence revealing the presence of polyphenolic compounds on the background of a possible double encapsulation process. In Figure 2b, the dyed variant 1 displayed irregularly shaped solsiform formations ranging from 56.79 to 139.32 µm. Within the formations, several spherosomes were identified, with sizes between 4.84 and 16.27 µm, that showed an emission in the red wavelength domain, most likely compounds represented by the black rice anthocyanins. In Figure 2d, in the dyed variant 2, the formed spherosomes presented significantly higher values between 5.39 and 37.91 µm. In both of the fluorophores dyed samples, the presence of anthocyanins was identified, highlighting the fact that the biologically active compounds are protected by the encapsulant matrix. 

### 2.4. In Vitro Cytotoxicity of the Microencapsulated Powders

**Figure 3:**
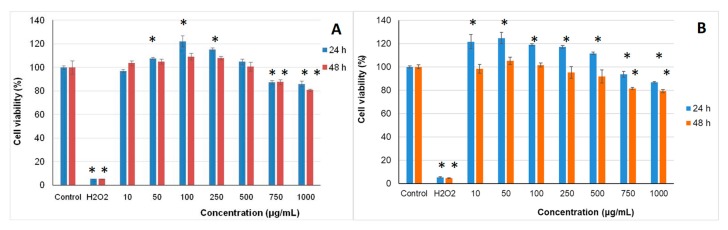
Cell viability of L929 fibroblasts cultivated in the presence of microencapsulated black rice extracts in variant 1 (**A**) and variant 2 (**B**) for 24 h and 48 h, respectively, determined by NR assay. The results are expressed as percent relative to the control culture (untreated), considered 100% viable. The values represent mean ± SD (*n* = 3). * *p* < 0.05, compared to control.

Release of possible toxic compounds from microencapsulated samples and cell viability were evaluated in L929 fibroblast cell culture by neutral red assay. The results showed that the microencapsulated variants, at all tested concentrations in the range 10–1000 µg/mL, were cytocompatible in L929 fibroblast culture, at 24 h and 48 h of cultivation (Figure 3). Concentrations between 50 and 250 µg/mL of variant 1 and 10 and 500 µg/mL of variant 2 stimulated cell proliferation, after 24 h of cultivation, compared to the control culture (untreated). Treatment with higher concentrations of microencapsulated powders (750–1000 µg/mL) induced a decrease in cell viability, but the values were higher than 80%, indicating they were not cytotoxic. 

**Figure 4:**
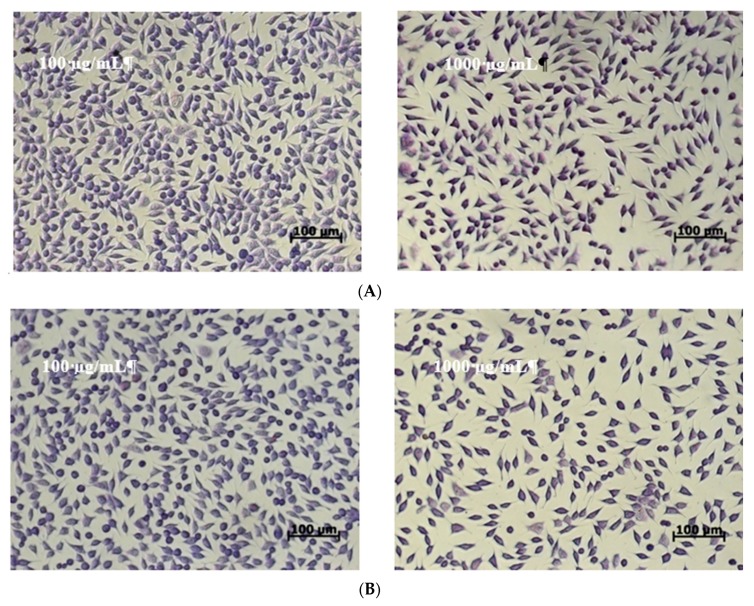
Light micrographs of L929 cells treated with microencapsulated powders: (**A**) variant 1 and (**B**) variant 2 for 48 h.

Cell morphology observations were in accordance with NR quantitative data (Figure 4). The images showed that the cells treated with microencapsulated extracts of black rice maintained their normal fusiform phenotype, characteristic for fibroblast cells, similar to the untreated culture. The cells were homogeneously distributed on the culture plate and their density was similar to that of the untreated culture. 

At concentrations of 50 to 250 µg/mL, the cell density was higher than that of the control culture. Higher concentrations of microencapsulated extracts decreased the cell density in treated culture plates. Similar studies have shown that anthocyanin-rich extracts of pigmented rice [19] were not toxic in normal cell culture, in the range of tested concentrations of 50 to 200 µg/mL.

Present results indicated a significant increase of the cytocompatibility domain after microencapsulation of extracts. Thus, the cell viability was higher than 80% for concentrations up to 1 mg/mL, especially in variant 2.

### 2.5. Thermal Stability of Anthocyanins in Microencapsulated Powder

**Figure 5:**
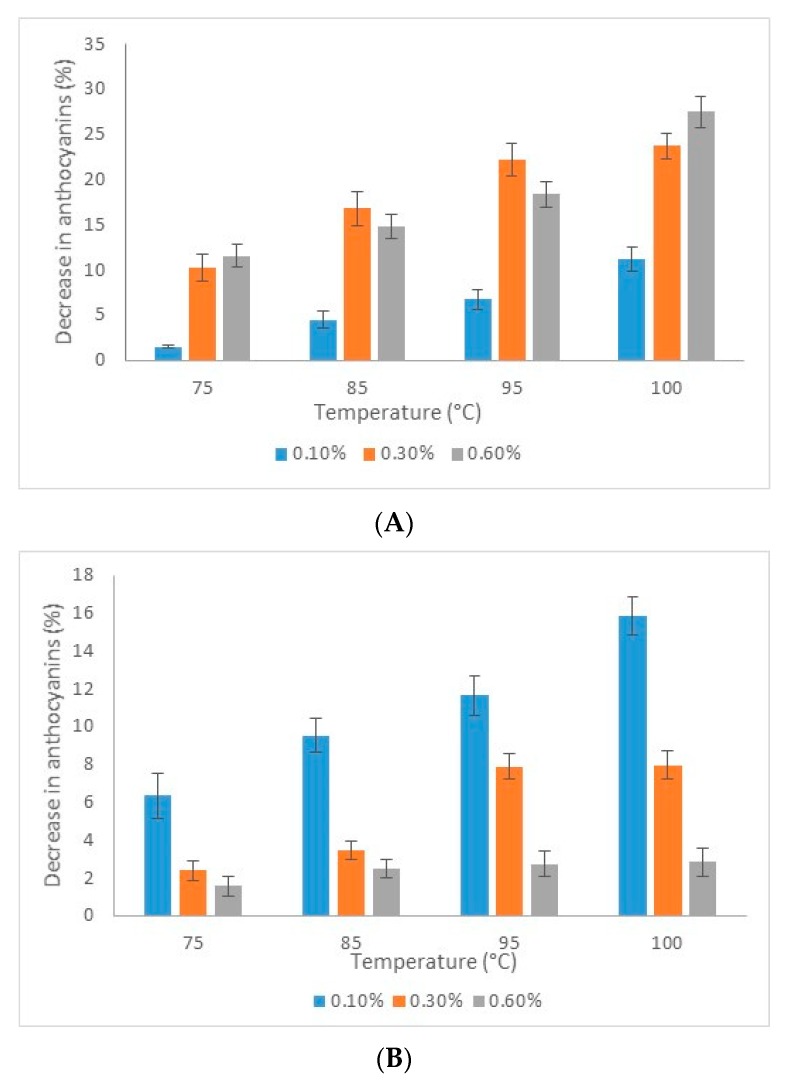
Evolution of the total anthocyanins content decrease in microcapsules obtained after thermal treatment at different temperatures varying between 75 and 100 °C for 15 min at different concentrations: (**A**) variant 1 and (**B**) variant 2.

Many studies have been performed on the stability of anthocyanins to heating, usually by treating the anthocyanin-containing solution in a water bath at 100 °C [20]. In the present study, the stability of anthocyanins was tested at different concentrations (0.1–0.6%) at temperatures ranging from 75 to 100 °C for 15 min. The effect of thermal treatment on the anthocyanins content in both variants are shown in Figure 5. 

In variant 1, at the lower temperature of 75 °C, the TAC decreased by 12% in solutions with higher concentrations, whereas increasing the temperature to 100 °C caused a decrease of 12% in solution with the 0.1% concentration of powder and by 24% and 28% in solutions with the 0.3% and 0.6% concentrations of powder, respectively. In variant 2, a higher stability with increasing concentration of the microencapsulated powder was observed. For example, heating at 100 °C caused a decrease in TAC, with 16% at 0.1%, 8% at 0.3%, and only 3% at 0.6%, respectively.

### 2.6. Formulation of Anthocyanins-Rich Pastry Cream

The pastry creams were analyzed for the TAC, TFC, TPC, and antioxidant activity and the results are expressed on the DW basis. At time t_0_, the C1 sample showed a TAC of 0.12 ± 0.03 mg C3G/g DW, TFC of 1.37 ± 0.77 mg CE/g DW, and TPC of 2.01 ± 0.10 mg GAE/g DW, whereas C2 presented a lower phytochemical content of 0.08 ± 0.006 mg C3G/g DW, 0.45 ± 0.23 mg CE/g DW, and 1.00 ± 0.13 mg GAE/g DW, respectively. Both products showed a satisfactory antioxidant activity of 40.55 ± 0.16 Mol and 42.30 ± 0.64 Mol Trolox/g DW.

The products were stored for 48 h at 4 to 6 °C, and the selected phytochemical and antioxidant activity were measured every 24 h. No significant variations were found in TFC and TPC, except a significant release of approximately 58% flavonoids in C2 after 48 h. Concerning TAC, both samples showed a decrease, with approximately 30% in C1 and 70% in C2. Both values showed satisfactory antioxidant activity around 43.00 mM Trolox/g DW during storage for 48 h at 4 to 6 °C.

## 3. Materials and Methods 

### 3.1. Materials 

Whey protein isolate (WPI) (protein content of 95%) was purchased from Fonterra (Auckland, New Zealand). Whey proteins hydrolysates (WPH) and bovine casein (CN) (95%) were purchased from the Hut Com. Ltd. (Manchester, UK). Catechin, 2,2-diphenyl-1-picrylhydrazyl (DPPH), 6-hydroxy-2,5,7,8-tetramethylchromane-2-carboxylic acid (Trolox), ethanol, sodium hydroxide, Folin-Ciocalteu reagent, and gallic acid were obtained from Sigma Aldrich (Steinheim, Germany). The black rice (*Oryza sativa* L) was purchased from a local market (Galati, Romania) in October 2018. 

### 3.2. Phytochemicals Extraction and Characterization 

For the extraction of phytochemicals, 500 g of black rice were mixed with 1000 mL of hot water (75 °C) and allowed to stand for 12 h. The supernatant was collected and used to estimate the phytochemicals content in terms of TAC, TPC, TFC, and antioxidant activity, as described by Turturică et al. [21].

### 3.3. Molecular Modelling Investigations

The in silico approach was used to check the interaction between milk proteins and major anthocyanins found in black rice, namely C3G and P3G. The three-dimensional models of alpha-S1 (αS1CN), alpha-S2 (αS2CN), beta (βCN), and kappa (kCN) caseins were prepared based on the amino acid sequences from P02662, P02663, P02666, and P02668, respectively, deposited in the UniProtKB database [22], by using the I-TASSER protein structure server [23,24]. Out of the five models generated by I-TASSER for each type of casein, the models with the highest confidence score (C-score) were selected for further docking studies (C-score of −2.18, −2.01, −2.35, and −2.42 for alpha-S1, alpha-S2, beta, and kappa casein, respectively). The molecular models of the main whey proteins, α-lactalbumin (αLA) and β-lactoglobulin (βLG), were obtained from the RCSB Protein Data Bank. The PDB IDs 1F6S of αLA [25] and 3NPO of βLG [26] were used in the study. Finally, the models of the C3G and P3G anthocyanins were built and optimized by means of HyperChem 8.0 software (Hypercube Inc., Waterloo, ON, Canada).

In order to check the interaction between the two types of compound used in the in silico study, molecular docking tests were carried out using the PatchDock algorithm [27], where the proteins were used as receptors for anthocyanin binding. For all protein–anthocyanin complexes, the docking models were scored based on the binding energy values and the top docking models were carefully analyzed in terms of interaction particularities using dedicated tools, such as LigPlot+ [28], PDBePISA [29,30], and VMD 1.9.3. [31].

### 3.4. Microencapsulation of Anthocyanins 

WPI, WPH, and CN were dispersed in black rice water extract. Different variants for microencapsulation were used, varying the ratio between WPI, WPH, and CN as follow: WPI:WPH:CN of 2:1:0.2 (variant 1) and 2:1:0.1 (variant 2), respectively. In order to ensure complete hydration, each variant was mixed at 650 rpm for two hours. After appropriate mixing, the pH of the samples was adjusted at pH 4.0. Further, the samples were frozen at −70 °C, and the ice crystals were then removed by freeze-drying (CHRIST Alpha 1-4 LD plus, Germany) at −42 °C under a pressure of 0.10 mBar for 48 h. Finally, the powders were collected and packed in metalized bags and stored in the freezer at −20 °C for later analyses. Each experiment was duplicated.

### 3.5. Powder Characterization

The powders’ characterization in terms of encapsulation efficiency, TAC, TFC, TPC, and antioxidant activity was performed as described earlier by Oancea et al. [32]. Briefly, the TAC was determined spectrophotometrically at 520 nm [33] and expressed as milligram of cyanidin-3-glucoside (C3G) equivalents/g DW. The colorimetric method based on the aluminum chloride capacity of forming stable acid complexes with flavanols was used to determine TFC (expressed as mg catechin equivalents CE/ g DW). The Folin–Ciocalteu method was used to determine the content of TPC (expressed as mg gallic acids equivalents GAE/ g DW). For the determination of antioxidant activity, the protocol for measuring antiradical activity on DPPH (2,2-diphenyl-1-picrylhydrazyl) was used and expressed as mMol Trolox/100 g DW. 

For the microencapsulation efficiency evaluation, the procedure described by Saénz et al. [34] was used, as the difference between the anthocyanins retention (AR) and the anthocyanins located in the microcapsule surface (AS). To quantify the AS, 200 mg of powder was mixed with 1 mL of ethanol and methanol (1:1). These dispersions were stirred at room temperature for 1 min and then centrifuged (4000× *g*, 10 min). For AR, 200 mg of powder were accurately weighed and dispersed in 1 mL of ethanol, acetic acid, and water (50:8:42). This dispersion was agitated using a Vortex (1 min) and then an ultrasonicator twice for 30 min. The supernatant was centrifuged at 20,000× *g* for 10 min and then filtered. TAC was quantified in supernatants by the pH-differential method and expressed as mg C3G/100 g DW. The microencapsulation efficiency (EE, %) was calculated with Equation (1):

(1) $$EE\%\;=\;\frac{\left(AR-AS\right)}{AR}\;\times \;100$$

### 3.6. Confocal Laser Scanning Microscopy

To assess the microstructure of the particles, a confocal laser scanning microscopy analysis was performed with a Zeiss Confocal Laser scanning system (LSM 710). The system is equipped with a diode laser (405 nm), Ar-laser (458, 488, 514 nm), DPSS laser (diode pumped solid state–561 nm), and HeNe laser (633 nm). The images were analyzed with the black edition of the ZEN 2012 SP1 software. To observe the fluorescence, the samples were observed in their native state for their natural autofluorescence and also dyed with two fluorophores (4′,6-diamidino-2-phenylindole, DAPI) (1 μg/mL) and Red Congo (40 μM), in a ratio of 3:1:1.

### 3.7. Cytocompatibility Testing of the Microencapsulated Powders

#### 3.7.1. Cell Culture and Treatment

Mouse fibroblasts from NCTC clone L929 cell line (ECACC, Sigma-Aldrich) were cultured in minimum essential medium (MEM) supplemented with 10% (*v*/*v*) fetal calf serum (FCS), 2 mM L-glutamine, and 1% (*v*/*v*) antibiotic mixture (penicillin-streptomycin-neomycin), in a humidified atmosphere with 5% CO_2_, at 37 °C, until subconfluence. 

Stock solutions of samples were prepared in culture medium, at a concentration of 1 mg/mL by incubation at 37 °C, for 24 h and then, they were filtered through 0.22-µm membrane filters (Millipore). Trypsinized cells were seeded in 96-well microplates, at a density of 4 × 10^4^ cells/mL and allowed to adhere by incubation at 37 °C, in humidified atmosphere with 5% CO_2_, for 24 h. Then, the culture medium was replaced with fresh medium containing different concentrations of samples ranging from 10 to 1000 μg/mL. The plates were incubated at 37 °C under standard conditions, for 24 and 48 h, respectively. Cells incubated in culture medium without sample were used as the negative control culture, while cells incubated with 0.003% (*v*/*v*) H_2_O_2_ served as a positive control. 

#### 3.7.2. Cell Viability

Cell viability was evaluated by neutral red (NR) assay, as previously described by Crăciunescu et al. [35]. Briefly, at the end of each incubation period, the culture medium was removed from each well and the cells were incubated with 50 μg/mL NR solution, at 37 °C, for 3 h. After cell washing, the incorporated dye was released in 1% (*v*/*v*) acetic acid solution in 50% (*v*/*v*) ethanol by gentle shaking, for 15 min. The amount of dye that was taken up was directly proportional to the number of viable cells. The optical density was measured at 540 nm in a Sunrise microplate reader (Tecan, Austria). The results are reported as the percentage relative to the control culture, considered 100% viable.

#### 3.7.3. Cell Morphology

Cell morphology after 48 h of cell incubation in the presence of samples was observed by light microscopy. Briefly, cells were washed, fixed in methanol, and stained with Giemsa solution. Micrographs were acquired using an optical microscope Axio Observer D1 equipped with a digital camera (Carl Zeiss, Germany).

### 3.8. Formulation of a Pastry Model Food (Cream for Cakes)

The two microencapsulated variants were used to formulate different model creams for cakes: C1 was prepared with variant 1, C2 with variant 2, and the control cream was prepared without encapsulated powder addition. The selected recipe listed: Sweet sour cream (35%), sugar (15%), and an amount of microencapsulated powders to reach the ratio of 0.5%. The microencapsulated powders were first mixed with the sugar and then added to the sweet sour cream. The mixture was mixed for 3 min using a domestic pin mixer. The creams were stored at 4 °C for further analysis. 

### 3.9. Statistical Analysis of Data

Unless otherwise stated, the data reported in this study represent the average of triplicate analyses and are reported as mean ± standard error of the mean. Statistical analysis of data was performed using one-tailed paired Student t-test. Significant differences were considered at *p* < 0.05.

## 4. Conclusions

In this study, whey protein isolate, whey protein hydrolysates, and casein were used for encapsulation of the anthocyanin-rich extract from black rice by freeze-drying, yielding high encapsulation efficiencies of almost 99%. Two dark-purple powders were obtained by varying the proportion of casein used as the encapsulation material. Single molecule level investigations indicated that the complexes formed between the main milk proteins and anthocyanins from black rice are stable from a thermodynamic point of view. The protein–anthocyanin complexes are mainly stabilized through hydrophobic interactions and hydrogen bonds, involving both the aglycon and glucosyl moiety of the encapsulated molecules. When characterizing the encapsulated powders, it was found that both variants presented elevated phytochemical contents, with significant antioxidant activity, which makes them valuable candidates for food applications as colorants and antioxidants. At all concentrations tested in our study, both microencapsulated variants were cytocompatible in the L929 fibroblast culture, and even stimulated cell proliferation after 24 h at concentrations up to 250 µg/mL for variant 1 and 500 µg/mL for variant 2. From a morphological point of view, the encapsulated powders showed the presence of bioactive compounds from black rice within some spherical particles with different dimensions, hence highlighting the possibility of a double encapsulation process. In order to facilitate the decision regarding incorporation in different food systems at the industrial level, the thermal stability of powders was tested at various concentrations and different temperatures. The results showed a good stability of powders to thermal treatment, with a significantly higher stability of anthocyanins at higher concentrations in the experimental variant with a lower casein content. The powders were tested in pastry cream in order to demonstrate the added value. Two variants of cream were obtained with significant antioxidant activity and a nice pink color, which remained unchanged during storage.

## Figures and Tables

**Table 1:** Details on the interaction between milk proteins (αLA-α-lactalbumin, βLG-β-lactoglobulin, alpha-S1 casein-αS1CN, alpha-S2 casein–αS2CN, beta casein βCN, and kappa casein-kCN) and main anthocyanins (cyanidin-3-*O*-glucoside and peonidin-3-*O*-glucoside).

Protein-Anthocyanins Complex	Interaction Energy, kJ/mol	Contact Area, Å^2^	ΔG^int^, kcal/mol	ΔG^diss^, kcal/mol	Residues Involved in Hydrophobic Contacts with the Anthocyanins	Residues Involved in Hb with the Anthocyanins
αLA-C3G	−172.59	560.70	−0.1	2.4	Thr^33^, Glu^49^, Phe^53^, Ile^59^, Asn^102^, Tyr^103^, Trp^104^, Ala^106^, Lys^108^	Thr^33^, Asn^102^, Ala^106^
βLG-C3G	−197.49	539.10	−0.3	2.6	Lys^69^, Ile^71^, Asp^85^, Ala^86^, Asn^88^, Asn^90^, Met^107^	Lys^69^, Ala^86^
βCN-C3G	−154.90	643.30	−0.5	2.8	Ile^41^, Lys^43^, Lys^44^, Ile^45^, Gln^104^, Pro^105^, Glu^106^, Val^107^, Pro^165^, Pro^167^, Leu^180^	Lys^43^, Lys^44^
αS1CN-C3G	−170.33	507.00	−0.8	3.1	Ser^56^, Ile^59^, Gly^60^, Glu^62^, Ser^63^, Leu^157^, Tyr^161^, Leu^164^, Phe^165^	Gly^60^
αS2CN-C3G	−187.69	561.20	−0.6	2.9	Leu^114^, Leu^176^, Asn^177^, Leu^179^, Ile^182^, Ser^183^, Gln^187^	Gln^187^
kCN-C3G	−181.25	677.50	0.0	2.3	Ala^106^, Lys^107^, Ser^108^, His^119^, Pro^120^, His^123^, Thr^154^, Pro^171^, Glu^172^, Val^173^, Glu^175^, Ser^176^, Pro^177^	Lys^107^, His^123^, Thr^154^, Glu^172^, Glu^175^
αLA-P3G	−179.98	487.60	−0.5	2.0	His^32^, Thr^33^, Val^42^, Asn^44^, Glu^49^, Tyr^103^, Trp^104^, Leu^105^, Ala^106^	Tyr^103^
βLG-P3G	−142.36	497.10	−0.1	1.6	Leu^39^, Val^41^, Leu^58^, Lys^69^, Ile^71^, Ile^84^, Asp^85^, Ala^86^, Leu^87^, Asn^90^, Glu^108^, Asn^109^	Asp^85^, Ala^86^, Glu^108^, Asn^109^
βCN-P3G	−232.18	676.20	−0.3	1.8	Lys^63^, His^65^, Tyr^75^, Pro^76^, Pro^78^, Glu^106^, Val^107^, Met^108^, Gly^109^, Val^110^, Ser^111^, Lys^112^, Leu^155^, Gln^156^, Met^159^, His^160^, Gln^161^	His^65^, Ser^111^, Gln^156^
S1CN-P3G	−219.83	485.80	−1.1	2.5	Ser^56^, Ile^59^, Gly^60^, Glu^62^, Ser^63^, Glu^65^, Leu^157^, Tyr^161^, Leu^164^, Phe^165^	Glu^65^
S2CN-P3G	−136.75	484.70	−0.7	2.2	Leu^121^, Pro^123^, Asp^125^, Val^127^, Arg^129^, Asn^130^, Val^154^, Glu^157^, Ser^158^	-
kCN-P3G	−200.86	656.10	−0.4	1.8	Pro^105^, Ala^106^, Lys^107^, Ser^108^, His^119^, Pro^120^, His^123^, Thr^154^, Asp^169^, Pro^171^, Glu^172^, Val^173^, Ile^174^, Glu^175^, Ser^176^	Ser^108^, His^123^, Asp^169^, Glu^172^, Ser^176^

ΔG^int^—solvation free energy gain upon assembly formation; ΔG^diss^—free energy of assembly dissociation; Hb—hydrogen bonds.
